# Semi-Autonomous Robotic Arm Reaching With Hybrid Gaze–Brain Machine Interface

**DOI:** 10.3389/fnbot.2019.00111

**Published:** 2020-01-24

**Authors:** Hong Zeng, Yitao Shen, Xuhui Hu, Aiguo Song, Baoguo Xu, Huijun Li, Yanxin Wang, Pengcheng Wen

**Affiliations:** ^1^School of Instrument Science and Engineering, Southeast University, Nanjing, China; ^2^State Key Laboratory of Bioelectronics, School of Instrument Science and Engineering, Southeast University, Nanjing, China; ^3^AVIC Aeronautics Computing Technique Research Institute, Xi’an, China

**Keywords:** brain–machine interface, gaze tracking, human–robot interface, continuous shared control, robotic arm reaching

## Abstract

Recent developments in the non-muscular human–robot interface (HRI) and shared control strategies have shown potential for controlling the assistive robotic arm by people with no residual movement or muscular activity in upper limbs. However, most non-muscular HRIs only produce discrete-valued commands, resulting in non-intuitive and less effective control of the dexterous assistive robotic arm. Furthermore, the user commands and the robot autonomy commands usually switch in the shared control strategies of such applications. This characteristic has been found to yield a reduced sense of agency as well as frustration for the user according to previous user studies. In this study, we firstly propose an intuitive and easy-to-learn-and-use hybrid HRI by combing the Brain–machine interface (BMI) and the gaze-tracking interface. For the proposed hybrid gaze-BMI, the continuous modulation of the movement speed via the motor intention occurs seamlessly and simultaneously to the unconstrained movement direction control with the gaze signals. We then propose a shared control paradigm that always combines user input and the autonomy with the dynamic combination regulation. The proposed hybrid gaze-BMI and shared control paradigm were validated for a robotic arm reaching task performed with healthy subjects. All the users were able to employ the hybrid gaze-BMI for moving the end-effector sequentially to reach the target across the horizontal plane while also avoiding collisions with obstacles. The shared control paradigm maintained as much volitional control as possible, while providing the assistance for the most difficult parts of the task. The presented semi-autonomous robotic system yielded continuous, smooth, and collision-free motion trajectories for the end effector approaching the target. Compared to a system without assistances from robot autonomy, it significantly reduces the rate of failure as well as the time and effort spent by the user to complete the tasks.

## Introduction

Assistive robotic systems have demonstrated high potential in enabling people with upper limb physical disabilities, such as traumatic spinal cord injuries (SCI), amyotrophic lateral sclerosis (ALS), and tetraplegic patients, to achieve greater independence and thereby increase quality of life (Vogel et al., 2015; Beckerle et al., 2017; Muelling et al., 2017). To produce the assistive robot control for severely impaired patients, the conventional manual control interfaces [computer mouse, keyboard, joystick, electromyography (EMG)-based interface, etc.] for able-bodied or mildly impaired people may no longer be applicable. This is because the aforementioned interfaces require that there are still some residual movements or muscle activities in the user. For people with no residual movement or muscular activity, previous studies have focused on two key aspects for facilitating the interaction between patients and the assistive robot. One is the design of human–robot interfaces (HRI). The other is the devising of human–robot coordination strategies tailored to the interface.

To provide HRI for individuals with severe upper extremity impairment, the brain signals or gaze signals have been largely explored through brain–machine interfaces (BMI) and gaze-trackers, respectively. With the advent of invasive BMI technology, the invasively recorded brain signals have facilitated successful manipulation of dexterous robotic arms (Collinger et al., 2013; Wodlinger et al., 2015) due to their high bandwidth and signal-to-noise ratio (SNR). Nevertheless, the benefit of effective robotic arm control may be outweighed by the medical risks associated with the current electrode implantation techniques. Non-invasive BMI, in particular the widely accepted electroencephalogram (EEG)-based BMI, provides a desirable alternative, and it is thus adopted in this study. However, it comes with a concomitant reduction in spatiotemporal resolution and effectiveness.

Different EEG paradigm-based BMIs have been employed to control the dexterous robotic arm. Since sufficient number of discrete user commands could be inferred with the steady-state visual evoked potential (SSVEP)-based BMI or P300-based BMI in theory, a lot of studies have utilized such BMIs to control the robotic arm (Perera et al., 2016; Hong and Khan, 2017; Qiu et al., 2017; Zhang et al., 2017; José de Jesús, 2018; Kumar et al., 2018; Chen et al., 2019; Duan et al., 2019; José de Jesús et al., 2019a, b). However, they involve flickering displays which may make some BMI user uncomfortable (Graimann et al., 2010). Besides, the user can only generate actions synchronously, resulting in a certain amount of time spent idle for users and thus slowing down the system. The motor imagery-based (MI-based) BMI, which does not depend on the external stimulus, allows for asynchronous control paradigms to move the robotic arm (Meng et al., 2016; Penaloza and Nishio, 2018). Nevertheless, the user has to switch many discrete MI states during the task; for instance, he/she needs to perform the left/right hand/both hands MI as well as both hands relaxing to move the end-effector leftward/rightward/upward/downward (limited discrete directions only) (Meng et al., 2016; Xia et al., 2017; Xu et al., 2019). In addition, the movement speed is usually not controlled or merely coarsely modulated by the signal’s time-resolved power. Such a control method will not only lead to decreased BMI classification accuracy as the number of classes increases, but it is also non-intuitive in motion control (these brain states are not directly related to the desired movement directions of the end-effector) and prone to greatly increasing the mental workload during the task (Yuan and He, 2014). In other words, none of the aforementioned studies using the multi-class MI-based BMI are able to implement an intuitive and effective interface capable of providing continuous-valued velocity (i.e., including the movement direction and speed simultaneously) control signals for a robotic device.

We envision continuous-valued velocity control signals to be advantageous for controlling the dexterous assistive robotic arm where a user could intuitively perform volitional control to change the robotic arm end-effector’s velocity, resulting in relatively smooth changes in position over time. Though recent studies show that the continuous-valued velocity of the upper limb could be decoded from the EEG signals with regression models via an MI paradigm (Yuan et al., 2010; Robinson and Vinod, 2016; Korik et al., 2018), the quality of the predicted hand movement parameters are still far away from the requirements for robotic arm manipulation applications in real world, not to mention that the intensive calibration phrase for collecting sufficient training data often lasts for more than half an hour (Kim et al., 2015).

Thereby, intuitive and easy-to-use interfaces that produce continuous-valued outputs while demanding less training are strongly desired. The gaze-tracking system may shed some light on building such an interface. In fact, gaze constitutes an intuitive input for continuous-valued positions in 2D control tasks (e.g., moving a cursor freely on a computer screen) without extensive training. However, one of the main limitations of gaze tracking is that the input may be intention-free (without necessarily selecting there), even though the user stares at somewhere on the computer screen. To this end, the hybrid gaze-BMI has been proposed to predict the web user’s click intention (Slanzi et al., 2017), explicit selection of the target in robot reaching (Frisoli et al., 2012), and ability to carry out grasping (McMullen et al., 2014; Zeng et al., 2017) and pushing tasks (Schiatti et al., 2017). In these studies, the continuous-valued position of an object is firstly indicated by the user in the 2D space through the gaze-tracking device, and this is then confirmed once the MI state is detected with a simple “MI vs. rest” two-class BMI. In our work, the hybrid gaze-BMI will be further extended to offer continuous-valued velocity control signals.

Along with the efforts of current studies to design intuitive and easy-to-use interfaces for motor-impaired people interaction with assistive robots, there are also endeavors made toward devising human–robot coordination strategies catering to specific applications, such as robotic arms and wheelchairs. In general, the noisy, non-stationary, and low-dimensional characteristics of control signals hinder the current interfaces to reliably issue commands in real-time for applications that require high precision and safety. To increase usability and reduce the cognitive burden of the user, the shared control strategies have been commonly adopted by adding autonomous supportive behavior to the system. According to the exact specification of how control is shared between the user and the autonomy, the existing shared control paradigms for human-robot interactions based on the interfaces can be generally divided into two lines.

One line of the paradigms triggers a fully pre-specified autonomous takeover when a specific mental state, e.g., the motor imagery (MI) state or a response state to a visual stimulus, is detected by BMI (McMullen et al., 2014; Beckerle et al., 2017; Zhang et al., 2017). We have recently presented a semi-autonomous robotic system with similar BMI triggers to initiate a subtask in a sequence (Zeng et al., 2017). While these systems are effective at accomplishing the task since a high amount of autonomy can outperform direct BMI user control, users instead preferred to have more control (Kim et al., 2012).

Another line of paradigms enables users to have more control with high-level user commands (e.g., movement directions of the end-effector or the wheelchair, etc.), while fully relying on the autonomy to generate the precise and safe low-level reactive robot motions (e.g., target approaching, collision avoidance, etc.). Researchers have exploited EEG signals for indoor navigation for a telepresence robot (Leeb et al., 2013) and the wheelchair (Zhang et al., 2016; Li Z. et al., 2017), combining left–right navigation signals with reactive robot behaviors to avoid obstacles. A system has been developed to enable users to specify a 2D end-effector path via a click-and-drag operation, and the collision avoidance is implemented with a sampling-based motion planner (Nicholas et al., 2013). The operator’s gaze is employed to indicate the target, and then the robotic arm is guided to reach the target, both by utilizing potential fields for autonomy (Webb et al., 2016). In the abovementioned paradigms, since the low-level robot motions are exclusively realized with the motion planner-based autonomy without the involvement of users, the user can regain the control authority only when the reactive behavior (e.g., collision avoidance) finishes (Kim et al., 2006). Studies have shown that users often report frustration when it becomes obvious that the system is providing autonomous control, reducing the sense of agency (Muelling et al., 2017). To the best of our knowledge, there are few attempts to investigate the simultaneous blend of autonomous control and the user control with non-invasive human–robot interfaces due to the fact that the user control commands are discrete-valued with these existing interfaces.

In this work, we present a semi-autonomous assistive robotic system that could be potentially used by severely motor-impaired people, for deliberate tasks involving the target reaching and obstacles avoiding. With this system, the user constantly utilizes his/her gaze and EEG signals to freely and intuitively direct the movement of the robotic limb end-effector while receiving the dynamical assistance from the robot autonomy. Our contribution is twofold. (1) In addition to the mode for discrete target selection with the previous hybrid gaze-BMI, we extend such hybrid interfaces toward a new mode for asynchronously providing continuous-valued velocity commands by which the user can retain continuous motion control of the end-effector. The proposed new mode constitutes an intuitive and easy-to-learn-and-use input, where the continuous modulation of the movement speed via the motor intention is simultaneous to the unconstrained movement direction control with the gaze signals in a seamless way. (2) Distinguished from previous shared control strategies for non-invasive driven assistive robots where the control authority switches discretely between the user and the autonomy, our shared control paradigm combines user input and the autonomy at all times with the dynamical combination regulation, and this is thanks to the continuous-valued velocity control via the new HRI. The paradigm is devised in this manner to maintain as much volitional control as possible while providing the assistance for the most difficult parts of the task. Although the idea of shared control is not new, the present study is to our knowledge, the first application of shared control to the assistive robotic arm driven by continuous-valued velocity based non-invasive hybrid gaze-BMI. The experiments are performed by a number of able-bodied volunteers, and the results show that the new HRI-driven semi-autonomous assistive robotic system allows for a continuous, smooth, and collision-free motion trajectory for the end-effector approaching the target, significantly reducing the rate of failure as well as time and effort spent by the user to complete the tasks.

## Materials and Methods

**FIGURE 1:**
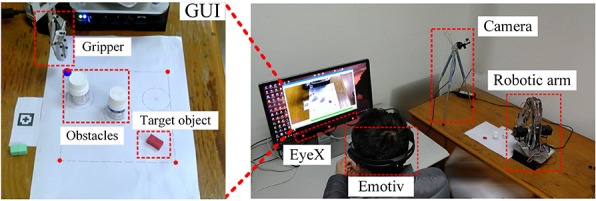
The overview of the experimental setup.

The experimental setup used in this study is depicted in Figure 1. The assistive robotic system consists of an eye-tracker, a device for recording EEG signals, a web camera, a robotic arm, a computer, and a monitor. As shown in Figure 1, a subject is seated in front of the monitor while controlling the movement of the robotic arm end-effector via the hybrid gaze-BMI. Before each task, the end-effector and the target (the red cuboid in Figure 1) were placed at two sides of the workspace. Two light obstacles (white cylinders in Figure 1) were set down in static locations between the target and the initial position of the end-effector throughout the experiments. In case the obstacle were to be knocked away by the end-effector in a run, it would then be relocated to the fixed coordinates in the next run. In our previous work, we investigated how to improve the robot grasping performance with the hybrid gaze-BMI control (Zeng et al., 2017). Thereby, we will focus on improving the reaching performance in the current study, and the grasping task will be completed automatically.

In specific, the reach-and-grasp task was divided into three stages. In stage 1, the user was to specify his intended target for the assistive robotic system, using the hybrid gaze-BMI operating in a discrete selection mode (refer to sub-section “Two operation modes of the hybrid gaze-BMI control”). Upon observing the virtual rectangle appearing around the target, the user got to know that the position of the target has been successfully communicated to the assistive robotic system. Subsequently, the system automatically switches the hybrid gaze-BMI into a continuous-velocity control mode (refer to sub-section “Two operation modes of the hybrid gaze-BMI control”). In stage 2, the user was to employ the hybrid gaze-BMI for moving the end-effector sequentially to reach the target across the horizontal plane parallel to the table while avoiding collisions with obstacles. Once the end-effector entered a pre-specified zone right above the target object (defined by a virtual cylindrical region centered above the target with a radius of 5 mm), it was forced to halt and hover over the target. In stage 3, the system executed a pre-programed procedure, i.e., the end-effector moved down, adjusted its gripper orientation according to the orientation of the target in the workspace, and grasped the object.

The design for the first two sequential stages took advantage of the natural visuomotor coordination behavior of human beings. Specifically, when a human decides to pick up an object, he/she usually first looks at the object, and then performs the hand reaching under the visual guidance. Moreover, following the suggestion in Meng et al. (2016), the original 3D-reaching task is accomplished by a combination of two sequential 2D-reaching tasks (i.e., the last two stages of tasks) in the horizontal plane and the vertical one, respectively. Such a design could effectively reduce the number of DoFs that the HRI has to provide, while it would still allow the user to reach the object in 3D space.

**FIGURE 2:**
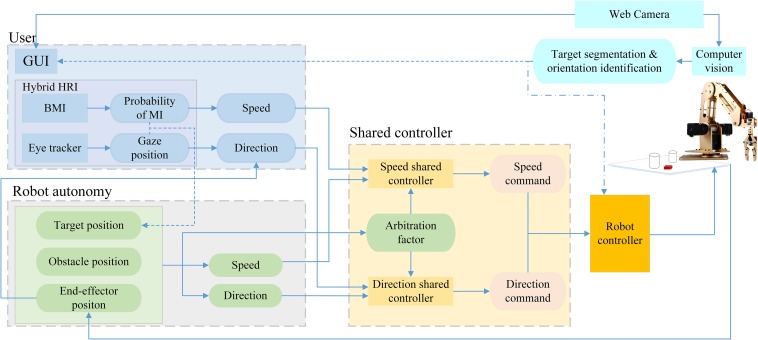
The block diagram of the proposed semi-autonomous robotic system. The dotted arrows denote the information flows only for Stage 1, and the dot-dashed arrow represents the information flow only for Stage 3.

Figure 2 introduces the block diagram of the proposed semi-autonomous robotic system, which consists of four main functional blocks:

(1) Hybrid Gaze-BMI, which combines gaze tracking and BMI. It firstly operates in a discrete selection mode for inputting the user’s intended target location in stage 1, and is then automatically switched to operate in a continuous-velocity control mode for inputting the user’s velocity commands to move the robotic arm end-effector horizontally toward the target in stage 2;
(2) Camera and Graphical User Interface (GUI), which provide the live scene of the robotic arm workspace for the normal and enhanced visual feedback as well as the coordinate transformations from camera coordinate system to the robot coordinate system for all the three stages. The Computer Vision implements the object segmentation and the object orientation identification for the target in stage 1 and stage 3, respectively;
(3) Shared Controller, which fuses the user commands from the hybrid gaze-BMI and the robot autonomy commands to form a new one, for directing the end-effector toward the target horizontally while avoiding obstacles in stage 2;
(4) Actuated System and Control, where the resulting end-effector commands are converted into reaching and grasping motions with a 5-Dof robotic arm.

The details about the individual modules of the system and the flow of information between them are described below.

### The Hybrid Gaze-BMI

#### Gaze Tracking

For the gaze tracking, a consumer-level desktop eye tracker, EyeX (Tobii AB Inc., Sweden), was employed. It did not require continuous recalibration and allows moderate head movements. The eye tracker was mounted at the bottom of the host PC monitor, it detects the user’s pupils and then projects the pupils onto the screen, i.e., the outputs of the eye tracker sensor system are the user’s gaze locations on the screen. The raw gaze data were transmitted to the computer via USB 3.0 at a sampling rate of 60 Hz. Since human eyes naturally make many involuntary movements including rolling, microsaccades, and blinking, the gaze signals acquired from the EyeX system were smoothened. In specific, a 10-point moving average filter is utilized to cancel out minor gaze fluctuations, while leaving performance on fast movements as unchanged as possible. Then, the filtered gaze points were fed to the shared control script every 30 ms.

#### Brain–Machine Interface

Given the final goal of developing affordable and usable assistive technology, a low-cost commercial EEG acquisition headset, Emotiv EPOC + (Emotiv Systems Inc., United States), is used to record the EEG signals. This device consists of 14 EEG channels (AF3, F7, F3, FC5, T7, P7, O1, O2, P8, T8, FC6, F4, F8, and AF4) according to the 10–20 system. The EEG signals are communicated to the host PC via Bluetooth with a sampling rate of 128 Hz.

In this study, we used the OpenVibe toolbox for the offline calibration of a 2-class BMI classification model and the online detection of the MI state. During the offline calibration phase, the EEG signals for the motor imagery state, and the rest state were recorded. Afterward, the segmented signals were bandpass-filtered between 8 and 30 Hz with a 5th-order Butterworth bandpass temporal filter. Subsequently, the commonly adopted spatial filtering method for the feature extraction in MI-based BMI, i.e., common spatial pattern (CSP), was applied on the signals. This was to find the directions that maximized variance for one class while minimized variance for the other class. The logarithms of the normalized power for the spatially projected signals were ultimately employed as the input features of the Bayesian linear discriminant analysis (LDA) classifier. The Bayesian LDA classifier assumed that the 2-class training data follow multivariate normal density distributions, and they had the same covariance matrix but different mean vectors and were estimated with equations below:

(1) $${\hat{\mu }}_{i}=\frac{1}{N_{i}}\sum_{x\in C_{i}}x,\left(i=0,1\right)$$

(2) $$\hat{\text{Σ}}=\frac{\sum_{k=1}^{N}\sum_{i=0}^{1}\left(x_{k}-{\hat{\mu }}_{i}\right)\,{\left(x_{k}-{\hat{\mu }}_{i}\right)}^{T}}{N-2}$$

where *x* represents a sample that belong to class “MI” (*i* = 1) or class “rest” (*i* = 0), *N*_*i*_ is the number of training samples belonging to class *i*, *C*_*i*_ denotes the set of training samples belonging to class *i*, *N* is total number of training samples, and “T” denotes the transpose. Then, the posterior probability value for the MI state was calculated by:

(3) $$P\left(x|y=i\right)=\frac{1}{\sqrt{2\,\pi \,\left|\hat{\text{Σ}}\right|}}\exp\left(-\frac{1}{2}\left(x-\mu_{i}\right){\hat{\text{Σ}}}^{-1}{\left(x-\mu_{i}\right)}^{T}\right),\left(i=0,1\right)$$

(4) $$P_{M\,I}=P\left(y=1|x\right)=\frac{P\left(x|y=1\right)P\left(y=1\right)}{\sum_{i=0}^{1}P\left(x|y=i\right)P\left(y=i\right)}$$

During the online phase, a 1 s-long sliding window in a step of 125 ms was used to update the feature values, and then the updated posterior probability value for the MI state was delivered to the shared control script on host PC through the VRPN protocol with the OpenVibe toolbox.

#### Two Operation Modes of the Hybrid Gaze-BMI Control

In this work, the hybrid gaze-BMI operated in two modes. In stage 1, as in Frisoli et al. (2012), McMullen et al. (2014), and Zeng et al. (2017), the user exploited the interface in a discrete selection mode to specify the intended target location for the system by firstly gazing at the center of the target object in GUI and then issuing the confirmation once the posterior probability value for the MI state exceeded the threshold (0.6 in our experiment). After a successful target object selection indicated by the augmented reality (AR) feedback (illustrated in subsection “Camera, GUI and Computer Vision”), the hybrid gaze-BMI automatically entered the continuous-valued velocity control mode in stage 2 (the horizontal reaching). With the hybrid interface in such a mode, the user had to constantly specify the sequential locations in GUI, to which he/she desires the end-effector to move, using the eye-tracker. At the same time, the user had to perform the motor imagery of pushing the end-effector with his/her dominant arm, and the 2-class BMI constantly produced the continuous-valued posterior probability for the MI state (ranging between 0 and 1), representing the detection certainty that the user entered the MI state. Such a unidimensional scalar index was then utilized to regulate the continuous-valued movement speed of the end-effector during the horizontal reaching task (detailed in subsection “The Proposed Shared Control Paradigm”).

### Camera, GUI and Computer Vision

A USB camera, with a resolution of 1,280 × 720 pixels, was placed on top of the setup to capture the live video of the robot workspace. It streams the horizontal view in robot coordinates system to the host PC via USB 2.0, and the video was displayed on the monitor with GUI. The user closes the loop by viewing video feedback and directing the end-effector accordingly.

**FIGURE 3:**
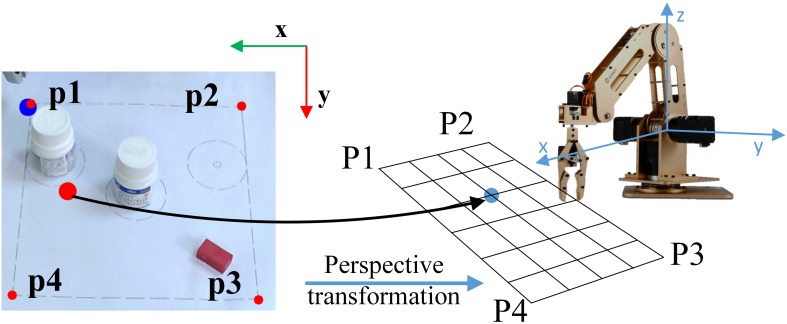
An illustration of mapping the camera’s coordinates to the robotic arm’s coordinates.

In order to select the target and control the movement direction of the end-effector, the system needs to know the position of the gaze points from GUI in the robot coordinates. An autonomous algorithm was implemented to build the mapping from the gaze coordinates on the screen to the robot coordinates. For this purpose, in the calibration phase (executed only once), four points were selected on the screen with known coordinates on the robot arm frame of reference. Then the identification of perspective transformation was accomplished with the 4-point getPerspective procedure from the OpenCV toolbox. Such a calibration is illustrated in Figure 3.

**FIGURE 4:**
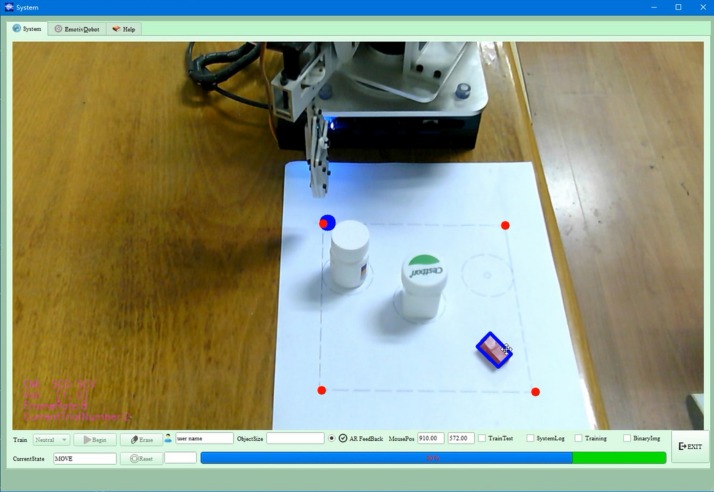
The selected target highlighted with a virtual rectangle frame surrounding it.

In stage 1, we provided the AR feedback to the user through the GUI to indicate the successful target object selection. Namely, as in our previous work (Zeng et al., 2017), the target (a cuboid in the current study) was highlighted with a virtual rectangle frame surrounding it (Figure 4) when the cursor (gaze point) was over the center of it and the motor imagery state was decoded from the BMI. Specifically, with the OpenCV and OpenGL toolboxes, the hybrid interface specified object was firstly segmented from the image took by the camera and was then it is overlaid with a virtual rectangle frame around.

In stage 2, since this paper mainly focus on the shared control method, the locations of obstacles in the workspace were static and known to the system, and the depth sensor was therefore not used to detect them in our paper.

In stage 3, to grasp the target (i.e., the cuboid) automatically, the orientation of the target had to be communicated to the robot system for adjusting its gripper pose. To this end, the orientation of the target was estimated by performing the geometric fitting of rectangles with a smoothly constrained Kalman fiter (Geeter et al., 1997).

### The Proposed Shared Control Paradigm

The devised shared-control paradigm consisted of a movement-speed shared controller and a movement-direction shared controller. In such shared controllers, the commands from the user and the robot autonomy were dynamically blended in order to generate the final velocity control commands for the end-effector sent to the robotic arm control system. The final velocity control command is written below:

(5) $$\vec{V_{f\,i\,n\,a\,l}}=S_{o}\,\vec{D_{o}}$$

where *S*_*o*_is the scalar speed of the robotic arm end-effector obtained from the speed shared controller, and $\vec{D_{o}}$ stands for the direction control command from the direction shared controller. These two shared controllers are described in detail in the following sub-sections.

#### The Speed Shared Controller

To achieve a continuous control of the speed of the robotic arm end-effector, the movement speed was modulated by the instantaneous strength of his/her dominant arm motor imagery state constantly detected by the BMI. Specifically, the speed of the end-effector was set to be proportional to the posterior probability assigned to the motor imagery state as follows:

(6) $$S_{h}=P_{M\,I}\,S_{m\,a\,x}$$

where *S*_*max*_ = 1.8cm/*s*represents the maximum speed of the robotic arm end-effector that can move, *P*_*MI*_denotes the posterior probability assigned to the motor imagery state using the Bayesian LDA classifier on EEG signals in a sliding window, and *S*_*h*_stands for the speed commands generated by the user. With increased level of motion intention, the user can complete a task at a higher speed. Undoubtedly, such an intermediate feedback will increase the involvement of the user during the task and maintain his/her sense of control. To avoid a sudden change in the movement speed of the end-effector, the output speed commands were filtered using a 2.5 s-long window in a step of 100 ms.

To further develop the human–robot blending of the movement speed commands, there were two issues to be addressed. One was to devise the assistance command provided by the robot autonomy, the other was the design of the arbitration scheme. Prior work indicated that users subjectively preferred the assistance when it lead to more efficient task completion (You and Hauser, 2011; Dragan and Srinivasa, 2013; Javdani et al., 2018). Thereby, to enable fast reaching, full speed was adopted for the assistance command from the robot autonomy. For the arbitration scheme, following the suggestions of previous user studies (Kim et al., 2006; Dragan and Srinivasa, 2013; Muelling et al., 2017), we decided to allow the user to directly control the majority of the movement and smoothly increase the assistance for realizing an efficient reaching, based on the system’s confidence of the estimated user’s intent to reach the target. More specifically, the arbitration between user commands and robot autonomy generated ones was realized by a linear blending function:

(7) $$S_{o}=\left(1-\alpha \right)\,S_{h}+\alpha \,S_{m\,a\,x}$$

**FIGURE 5:**
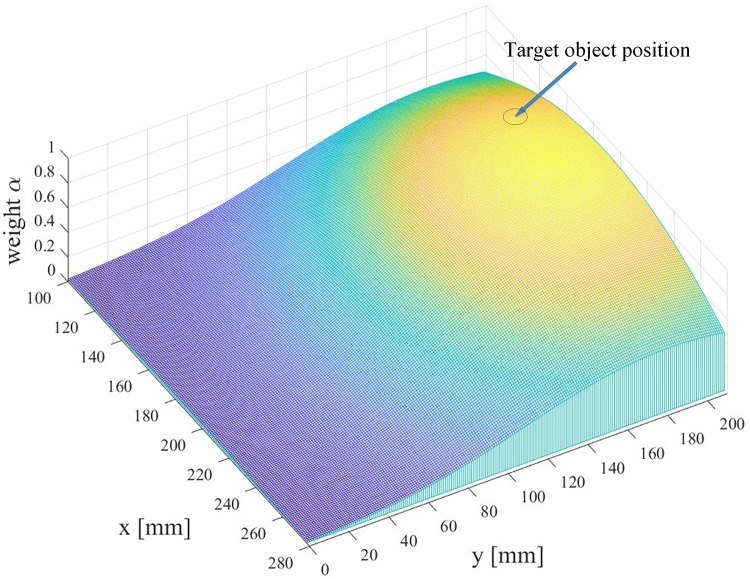
The distribution of the arbitration factor α.

Here, α represents the dynamical arbitration factor, defining the amount of assistance provided by the robot autonomy. It was calculated using a sigmoid function to enable smooth and continuous blending between the user and robot autonomy command:

(8) $$\alpha =\frac{1}{1+e^{-a\,\left(x_{d}-c\right)}}$$

where x_*d*_ denotes the distance from the robotic arm end-effector to the position of the target object on the horizontal 2D plane parallel to the table, *a* = −0.4 is a constant parameter, and *c* defines the distance so that α = 0.5. Figure 5 depicts the distribution of the arbitration factor α. According to Figure 5, as the end-effector moves closer to the target object, the certainty of user intention increases, the robot autonomy’s command gains more control weight, and then the end-effector approaches the target object more quickly. Nevertheless, if the user directs the end-effector to certain point far away from the target, the user regains the complete control of the robotic arm.

#### The Direction Shared Controller

**FIGURE 6:**
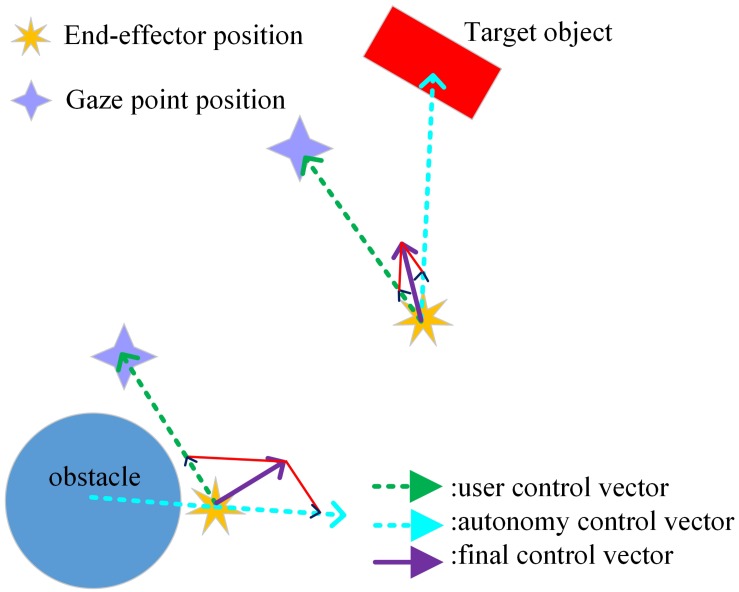
The principle for the shared control in direction.

For the direction control of the end-effector, a unit directional vector pointing from the end-effector to the user’s gaze position is derived as the user specified movement direction command, as shown in Figure 6. If the robot is controlled by the user alone, during the experiments, the user must always focus his/her attention to ensure control precision and collision avoidance, leading to heavy mental workload. Therefore, to reduce the difficulty, we provided assistance for the user, deriving from the robot autonomy. In specific, the robot autonomy also generated the direction control command based on the relative position between the robotic arm end-effector and the obstacle/target object, as shown in Figure 6. For the arbitration scheme, like that for the speed shared controller, we kept the user in control to the largest extent possible. Meanwhile, the robot autonomy gradually assisted the user by enforcing an attraction toward the target as well as the collision avoidance, when it became confident on the estimated user’s intent to reach the target or avoid obstacles. Specifically, the final direction control command sent to the robot are calculated using a linear blending of the user and the robot autonomy commands:

(9) $$\vec{D}=\left(1-\beta \right)\,\vec{D_{h}}+\beta \,\vec{D_{r}},\vec{D_{o}}=\frac{\vec{D}}{\left|\vec{D}\right|}$$

(10) $$\beta =\frac{1}{1+e^{-b\,\left(x_{d}-d\right)}}$$

where $\vec{D}$ is the norm of $\vec{D}$,$\vec{D_{h}}$ is the unit 2D directional vector generated by the user:

(11) $$\vec{D_{h}}=\frac{\vec{d\,g}}{\left|\vec{d\,g}\right|}$$

$\vec{d\,g}$ represents the 2D directional vector pointing from the end-effector to the gaze point. $\vec{D_{r}}$ is the unit 2D directional vector generated by the robot autonomy, either pointing toward the target or away from the obstacle:

(12) $$\vec{D_{r}}=\frac{\vec{d\,m_{o\,b\,j}}}{\left|\vec{d\,m_{o\,b\,j}}\right|}\,o\,r\,\frac{\vec{m_{o\,b\,s}\,d}}{\left|\vec{m_{o\,b\,s}\,d}\right|}$$

**FIGURE 7:**
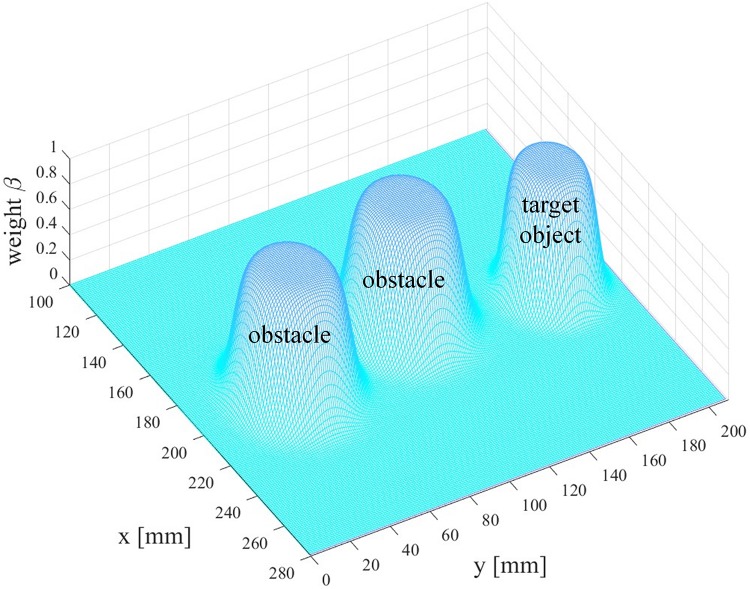
The distribution of the arbitration factor β.

$\vec{d\,m_{o\,b\,j}}$ denotes the 2D directional vector pointing from the end-effector to the target object, while $\vec{m_{o\,b\,s}\,d}$ is the 2D directional vector pointing from the obstacle to the end-effector. β is the arbitration factor defining the level of assistance provided by the autonomous system and is again calculated using a sigmoid function given in (10) to enable smooth and continuous blending. In the direction controller, the constant parameter *b* is set to −0.55, and *d* = 25 defines the distance so that β = 0.5, x_*d*_represents either the distance between the robotic arm end-effector and the obstacle or that between the end-effector and the target object. The distribution of the arbitration factor β is shown in Figure 7. As can be seen from Figure 7, when the user drives the end-effector close to the obstacle or the target, the robot autonomy gets more and more confident about the user’s intent to reach the target or avoid obstacles, and then it influences the robot end-effector more strongly than the user.

### Actuated System and Control

A proof-of-concept implementation of the proposed semi-autonomous robotic system was carried out using a 5-Dof robotic arm (Dobot Arm, Shenzhen Yuejiang Technology Co Inc., China). The robotic arm control system could automatically determine the joint motion commands based on the specified 3D positions of the end-effector using inverse kinematics. The developers also could specify the orientation of the gripper in order to grasp an object with a certain orientation in the workspace. The robotic arm control system communicated with the host PC through Bluetooth, receiving the input from the shared controller and sending the state parameters of the robotic arm to the host PC every 100 ms.

## Experiments

### Subjects and Tasks

Ten participants (eight males and two females, 25.2 ± 0.8 years old) were recruited from the campus to perform the objects manipulation tasks using the proposed HRI driven semi-autonomous robotic system. The study was approved by the Ethics Committee of Southeast University. Written informed consent was obtained from each subject.

The task was the 3-stage reach-and-grasp one introduced in detail at the beginning of section “Materials and Methods.” The user was to firstly select the target in stage 1 and then reach the target horizontally while avoiding obstacles in stage 2, using the hybrid gaze-BMI operating in a discrete selection mode and a continuous velocity control model in these two stages, respectively. In stage 3, the end-effector executed moving down, adjusting its gripper orientation and grasping the target and all in an automatic way.

### Offline Calibration Session

Operative tests were preceded by a calibration session for both the eye-tracker and the BMI.

Firstly, the built-in calibration procedure for the Tobii eye tracker EyeX was performed. It lasted less than 1 min for each subject, during which the user gazed at seven calibration dots sequentially appeared on the monitor.

Secondly, the BMI decoding model was trained for each subject with the offline calibration procedure described in sub-section “Brain-machine Interface.” Specifically, for the recoding of the motor imagery state, the user had to focus on observing the robotic arm end-effector’s predefined motion in the horizontal plane through GUI while imagining to push the end-effector with his/her dominant arm at the same time. For the rest state, the robotic arm did not move, and the user was asked to relax and avoid moving. The training session for each subject was composed of a randomly sorted sequence of 40 trials, 20 for the hand motor imagery tasks and 20 for the relax tasks. The execution of each task lasted for 4 s, and it was spaced from the beginning of the next task with an interval lasting randomly from 1 to 3 s during which the subject could relax concentration. Each task was triggered through visual cues developed with the openVibe toolbox and displayed in GUI. The data acquired during the training session were used to build the 2-class BMI decoding model composed of CSP and Bayesian LDA. The duration of the BMI calibration usually did not exceed 5 min. However, it was difficult to report the testing performance for the BMI decoder built with all the training data in our experimental setting. Thereby, we reported the fivefold cross-validation (CV) BMI decoding performance instead, which could to some extent reflect the performance for the BMI decoder built with all the training data. In the fivefold CV, when the posterior probability for the MI state exceeded 0.6, the mental state was classified to be MI; otherwise it was determined to be the “rest” state.

Before the formal online evaluation, the online decoding model of BMI was obtained by training with all the data from the offline calibration session mentioned above. Subsequently, a rehearsal phase was further launched for the purpose of familiarizing each user with the hybrid HRI-based robotic arm control, and this lasted less than 5 min for each of the 10 subjects. In the end of this phase, most of them could deliberately specify the intended target in stage 1 and constantly fixate gaze on any point on the screen to specify his/her desired movement direction for the end-effector while simultaneously regulate his/her strength of the MI state to modify the speed of the end-effector in stage 2.

### Online Evaluation Session

The main focus of this study was to apply the blending-based shared control for the robotic arm reaching driven by the proposed continuous-velocity hybrid gaze-BMI (i.e., the stage 2). Thereby, to evaluate the effectiveness of the proposed shared control paradigms for such an interface, the reaching tasks with or without shared control were conducted. Specifically, each subject executed 40 reaching trials with the following four types of control paradigms:

**SC***_*DS*_***:** The shared control both in speed and direction (i.e., *S*_o_ = (1−α)*S*_h_ + α*S*_*max*_ and $\vec{D}=\left(1-\beta \right)\,\vec{D_{h}}+\beta \,\vec{D_{r}}$, $\vec{D_{o}}=\frac{\vec{D}}{\left|\vec{D}\right|}$).

**SC***_*S*_***:** The shared control in speed only (i.e., *S*_o_ = (1−α)*S*_h_ + α*S*_*max*_ and $\vec{D}=\vec{D_{h}}$, $\vec{D_{o}}=\frac{\vec{D}}{\left|\vec{D}\right|}$).

**SC***_*D*_***:** The shared control in direction only (i.e., *S*_o_ = *S*_h_ and $\vec{D}=\left(1-\beta \right)\,\vec{D_{h}}+\beta \,\vec{D_{r}}$, $\vec{D_{o}}=\frac{\vec{D}}{\left|\vec{D}\right|}$).

**MC:** The manual control by the hybrid Gaze-BMI without any assistance from the robot autonomy.

There were 10 trials executed with **SC***_*DS*_*, **SC***_*S*_*, **SC***_*D*_*, and **MC**. The four paradigms were applied in a random order for the 40 trials, and the current control paradigm was not told to the user, in order to minimize the learning effect. For each online trial, the participant attempted to move the robotic arm end-effector sequentially toward the target in the horizontal plane while avoiding obstacles along the path with the proposed continuous-velocity control-based hybrid gaze-BMI. The robotic arm released the target and returned to the initial place of the workspace at the beginning of each run. The subject had a rest whenever needed between two trials. We did not conduct experiments with the previously proposed semi-autonomous robotic systems for performing the reach-and-grasp task, but the advantages of two key components in our semi-autonomous robotic system (i.e., the currently proposed hybrid Gaze-BMI and shared control paradigms) over the previous ones are illustrated in the section “The Proposed Hybrid Gaze-BMI” and section “The Proposed Shared Control Paradigms.”

### Evaluation Metrics and Statistical Analysis

To evaluate the effectiveness of the proposed-direction shared controller, the successful reaching rate (SRR) and the end-effector trajectory length (EETL) were acquired on the trials with (i.e., **SC***_*DS*_* and **SC***_*D*_*) and without (i.e., **SC***_*S*_* and **MC**) the movement-direction shared controller being applied. A successful reaching trial was defined as one during which the end-effector did not collide with obstacles before entering the pre-grasping zone. Then, the SRR identified the proportion for successful reaching trials out of the 10 trials applied with the four paradigms, measuring the effectiveness in improving the robot control precision for achieving the user’s goal. The EETL measured the user’s efforts to achieve the goal.

To evaluate the effectiveness of the proposed speed shared controller, the completion time (CT) was obtained on the trials applied with (i.e., **SC***_*DS*_* and **SC***_*S*_*) and without (i.e., **SC***_*D*_* and **MC**) the movement-speed shared controller being applied. CT measured the how long it took the subject to enter the pre-grasping zone, reflecting the efficiency in achieving the user’s goal.

A *p*-value of 0.05 was selected as the threshold for studying the statistical significance of those metrics. For metrics measured per trial (i.e., EETL and CT), we averaged the data across all ten trials for each paradigm, enabling us to treat each user as one independent datapoint in the statistical analysis. A Friedman test was used to assess whether each metric had a significant main effect among different paradigms since the data did not pass the test of normality (Jarque–Bera test). If a significant main effect was found, we further conducted the Tukey’s honestly significant difference *post hoc* test for multiple comparisons.

## Results

### The Offline Classification Performance of the BMI

The fivefold cross-validation classification accuracy of the BMI for each subject is shown in Table 1. The average recall for the relax state was 81.7 ± 6.8% (mean ± standard deviation) while that for the motor imagery state was 82.5 ± 5.6% (mean ± standard deviation). The overall average classification accuracy across the subject was 82.1% with a standard deviation of 4.8%. The highest fivefold CV classification accuracy of the 2-class BMI achieved was 90.3% with the data from subject 6, while subject 7 obtained the lowest performance with an average accuracy of 71.6%. Such offline classification performance was in line with the existing BMI studies (Martinezleon et al., 2016; Schiatti et al., 2016) using a Emotiv EPOC headset. It indicated that the adopted BMI calibration method could provide an applicable BMI decoder for our experiments.

**TABLE 1:** The fivefold cross-validation BMI classification performance for each subject.

**Participant ID**	**Recall (%)**		**Accuracy (%)**
	**Relax**	**Motor imagery**	**Total**
S1	81.6	82.9	82.3
S2	79.1	86.7	82.9
S3	90.2	76.5	83.4
S4	79.1	77.6	78.3
S5	93.1	81.9	87.5
S6	88.8	91.8	90.3
S7	70.4	72.7	71.6
S8	73.5	89.3	81.4
S9	81.2	84.2	82.7
S10	80.1	80.9	80.5
Mean ± STD	81.7 ± 6.8	82.5 ± 5.6	82.1 ± 4.8

### The Online Evaluation Performance

#### The Effectiveness of the Direction Shared Controller

The SRRs for the 10 subjects are listed in Table 2. It can be observed that, with the **SC***_*DS*_* or **SC***_*D*_* being applied, every subject attained 100% successful reaching rate. By contrast, with the **SC***_*S*_* shared control paradigm, the average successful reaching rate across subjects only achieved 67%, and the SRR of subject 5/subject 7 was the lowest (50%) among the 10 participates. The **MC** paradigm yielded an SRR of 66% with subject 7 being the lowest (40%). The Friedman test showed that SRR had a significant main effect (*p* ≪ 0.05), and the *post hoc* analysis revealed that the direction shared controller resulted in significant differences of SRR (**SC***_*DS*_* vs. **SC***_*S*_*, *p* = 0.0037, **SC***_*D*_* vs. **SC***_*S*_*, *p* = 0.0037, **SC***_*DS*_* vs. **MC**, *p* = 0.0009 and **SC***_*D*_* vs. **MC**, *p* = 0.0009). In a word, a subject completed the reaching task in all the trials without knocking against the obstacles when assisted by the direction shared controller, but easily failed in trials unassisted by such a direction shared controller.

**TABLE 2:** Number of trials with collisions and successful reaching rate in the experiments for the four control paradigms.

**Participant ID**	**Number of trials with collisions**				**Number of collision-free trials**				**SRR(%)**			
	**SC*_*DS*_***	**SC*_*D*_***	**SC*_*S*_***	**MC**	**SC*_*DS*_***	**SC*_*D*_***	**SC*_*S*_***	**MC**	**SC***_*DS*_*	**SC*_*D*_***	**SC*_*S*_***	**MC**
S1	0	0	4	5	10	10	6	5	100	100	60	50
S2	0	0	3	2	10	10	7	8	100	100	70	80
S3	0	0	4	3	10	10	6	7	100	100	60	70
S4	0	0	4	3	10	10	6	7	100	100	60	70
S5	0	0	5	5	10	10	5	5	100	100	50	50
S6	0	0	4	4	10	10	6	6	100	100	60	60
S7	0	0	5	6	10	10	5	4	100	100	50	40
S8	0	0	2	2	10	10	8	8	100	100	80	80
S9	0	0	0	1	10	10	10	9	100	100	100	90
S10	0	0	2	3	10	10	8	7	100	100	80	70
Mean	0	0	3.3	3.4	10	10	6.7	6.6	100	100	67	66
*p*-value **(SC*_*DS*_*** vs. **SC*_*S*_*)**									0.0037			
*p*-value **(SC*_*D*_*** vs. **SC*_*S*_*)**									0.0037			
*p*-value **(SC*_*DS*_*** vs. **MC)**									0.0009			
*p*-value **(SC*_*D*_*** vs. **MC)**									0.0009			

**FIGURE 8:**
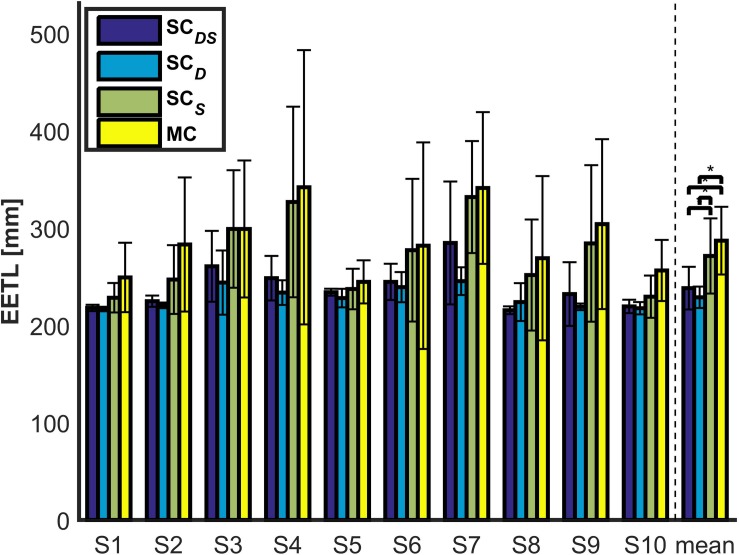
The EETLs for each subject and across subjects (the across-subjects performance difference with statistical significance is marked by “*”, *p* < 0.05).

The EETLs for each subject and across subjects are presented in Figure 8. One can observe that EETLs obtained with **SC***_*DS*_* or **SC***_*D*_* were generally shorter than those obtained with **SC***_*S*_* and **MC**. The Friedman test indicated that EETL differed significantly between such four shared control paradigms (*p* < < 0.05). The *post hoc* test showed that the direction shared controller led to significant differences of EETL (**SC***_*DS*_* vs. **SC***_*S*_*, *p* = 0.037, **SC***_*D*_* vs. **SC***_*S*_*, *p* = 0.0001, **SC***_*DS*_* vs. **MC**, *p* = 0.0016 and **SC***_*D*_* vs. **MC**, *p* < 0. 0001). The shorter path lengths during the movement-direction shared control trials suggested that the yielded movements were more direct, leading to reduced user efforts.

**FIGURE 9:**
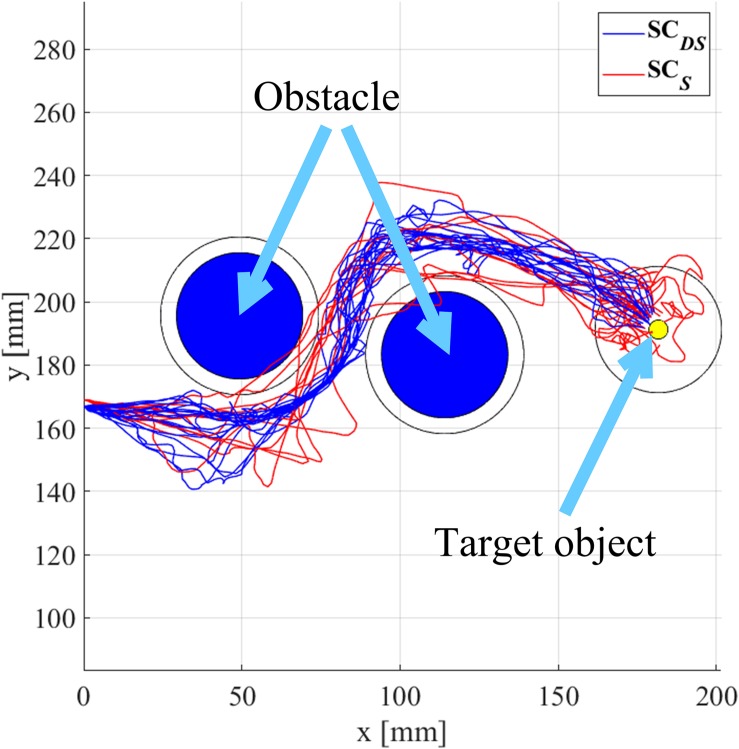
Trajectories of the robotic arm end-effector in the horizontal plane during the reaching task with or without the direction shared controller (subject 6).

In Figure 9, we provide a representative comparison of performance with direction shared control (**SC***_*DS*_*) and without direction shared control (**SC***_*S*_*) by plotting the trajectories of the robotic arm end-effector from all the trials for subject 6. The trajectories obtained with **SC***_*D*_* were not shown since there was no statistical difference in EETL between **SC***_*DS*_* and **SC***_*D*_* (*p* = 0.1733), and neither were the trajectories with **MC** as the differences in EETL between **SC***_*S*_* and **MC** (*p* = 0.3069) were not significant. The two circles in blue denote the obstacles along the reaching path, and the yellow circle is the target object. The red lines represent the trajectories that were generated with **SC***_*S*_* while the blue lines were those generated with **SC***_*DS*_*. From Figure 9, we observed that the reaching trajectories generated with assistance from the direction shared controller were smoother and more direct than those without. It also showed that there were no collisions between the end-effector and the obstacles with **SC***_*DS*_*. By contrast, the end-effector bumped against the obstacles for several trials with **SC***_*S*_*. Furthermore, when the robotic arm end-effector was not far from the target object, it entered the target object pre-grasping zone more directly with **SC***_*DS*_* than with **SC***_*S*_*. In other words, the movement direction shared controller improved task performance mainly by stabilizing the movement near the target or obstacles to eliminate unintended or inaccurate movements that could interfere with the task completing.

**FIGURE 10:**
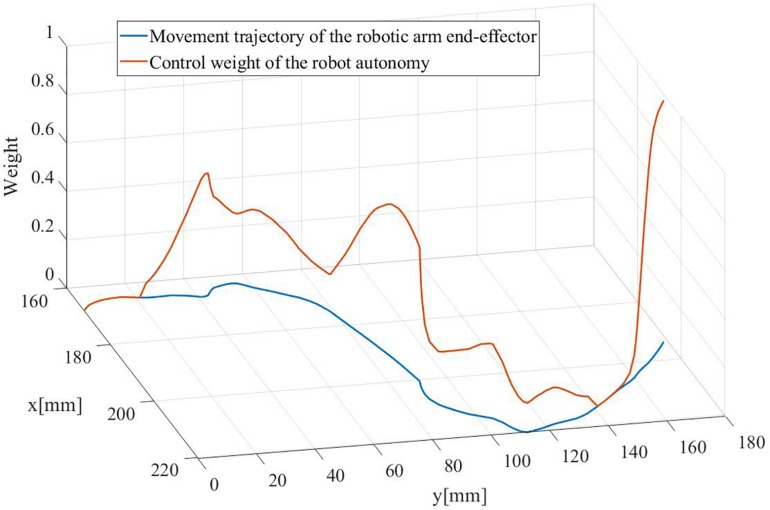
The evolving control weight for the robot autonomy in the direction shared controller during the 9th trial executing with **SC***_*DS*_* for subject 6.

Figure 10 shows the evolving control weight for the robot autonomy in the direction shared controller during the 9th trial that executed with **SC***_*DS*_* for subject 6. When the end-effector got closer and closer to obstacles, the control weight for the robot autonomy increased sharply. Consequently, the robot autonomy generated commands for the movement direction dominated the control role for enforcing an effective collision avoidance. As the end-effector was near to the target, the robot autonomy generated commands for the movement direction to dominate the control role again to enable a direct approaching toward the object.

#### The Effectiveness of the Speed Shared Controller

**FIGURE 11:**
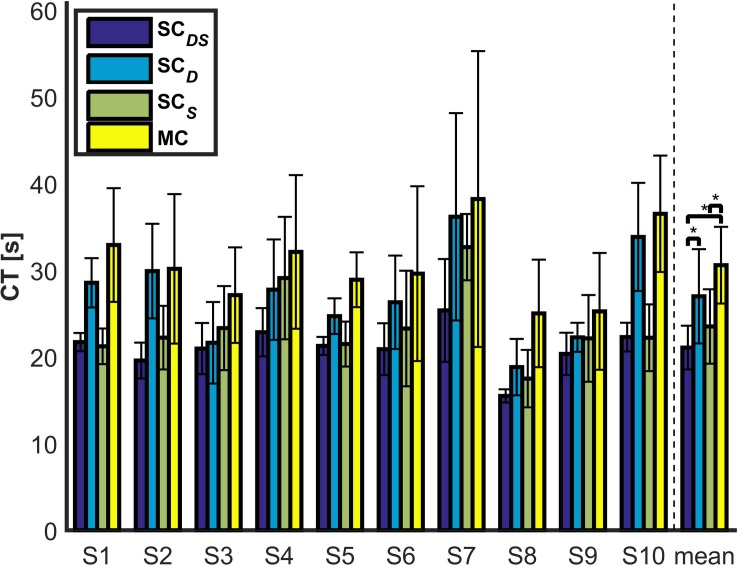
The CTs for each subject and across subjects (the across-subjects performance difference with statistical significance is marked by “*”, *p* < 0.05).

Figure 11 depicts the CT obtained with **SC***_*DS*_*, **SC***_*D*_*, **SC***_*S*_*, and **MC** for each subject and across subjects during the reaching task. In general, Figure 11 demonstrated that the **MC** paradigm was the slowest among the four paradigms. The Friedman test showed that CT had a significant main effect (*p* = 0.0017). The difference of CT between **SC***_*DS*_* and **MC** was statistically significant (*p* < 0.001). Furthermore, for the two paradigms that yielded 100% SRR (i.e., **SC***_*DS*_* and **SC***_*D*_*), the difference of CT between them was also statistically significant according to the *post hoc* analysis (*p* = 0.001), and all the subjects finished the object reaching task faster with **SC***_*DS*_* being applied than with **SC***_*D*_* being applied. In other words, the speed shared controller lead to improved efficiency of the reaching tasks.

**FIGURE 12:**
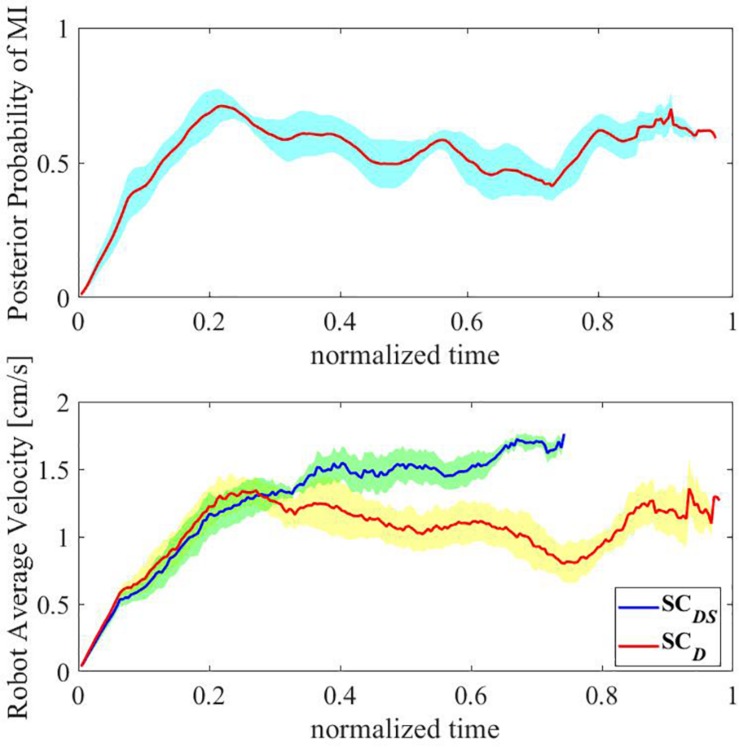
The evolving posterior probability values calculated by the BMI and the evolving speeds of the end-effector with **SC***_*DS*_* and **SC***_*D*_* in a normalized time scale (subject 1).

Recall that **SC***_*DS*_* and **SC***_*D*_* resulted in similar satisfying EETLs without a statistically significant difference, the difference of CT between them can be only attributed to their difference in speed during the reaching tasks. Moreover, since the direction shared controller was unable to modify the speed of the end-effector, the difference in speed can only be explained by whether the speed shared controller was applied or not. To provide a representative comparison of speed between **SC***_*DS*_* and **SC***_*D*_* (i.e., with speed shared control vs. without speed shared control), Figure 12 shows the evolving posterior probability values calculated by the BMI for subject 1 as well as the speeds of the end-effector with **SC***_*DS*_* and **SC***_*D*_*, both on a normalized time scale. The blue line in the top subfigure of Figure 12 denotes the evolving mean posterior probability value of the motor imagery state. In the bottom subfigure of Figure 12, the red line and the blue line represent the evolving mean speed values of the end-effector obtained with **SC***_*D*_* and **SC***_*DS*_*, respectively. The range of standard deviations is indicated with shaded background in the two subfigures of Figure 12. In our design, the continuous-valued output signals of the BMI Bayesian LDA classifier were used to linearly modulate the speed of the end-effector. According to the experimental results shown in the bottom subfigure of Figure 12, the evolving trend of the mean speed without assistances from the robot autonomy (i.e., mean speed obtained with **SC***_*D*_*) was indeed found to be generally aligned with that of the posterior probability value of the MI state during the reaching task. In the beginning of the trials (less than 5 s), similar low end-effector speeds were maintained for trials with shared control as the unassisted trials. After a while, the probability values assigned to the MI state became unstable, possibly due to the noisy and non-stationary characteristics of the brain signals. As a consequence, it led to unstable motion speed for the end-effector in unassisted trials. By contrast, when the robot autonomy assistance was provided, the end-effector speed remained much more stable with a slow increasing trend. Such a smooth speed profile was achieved by the dynamic speed compensation according to the certainty of the inferred user intention for reaching the target.

**FIGURE 13:**
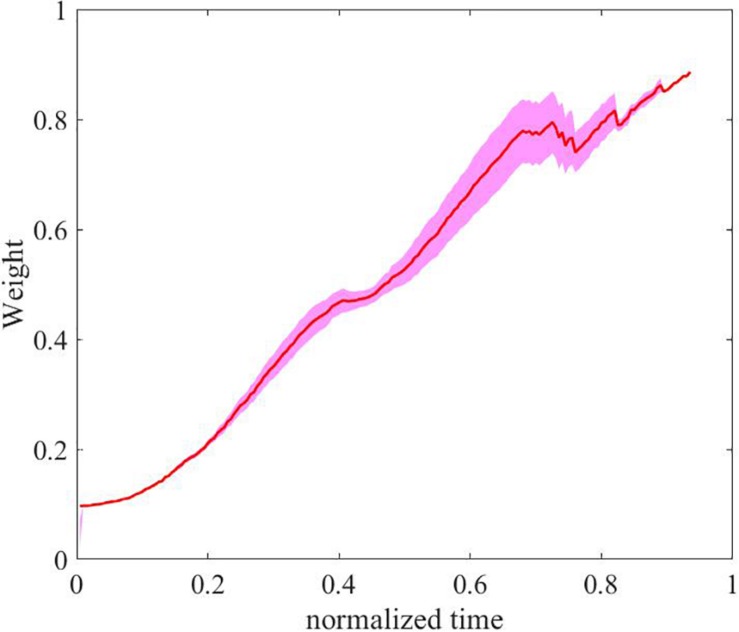
The arbitration factor for the robot autonomy from the speed shared controller in a normalized time scale.

For illustrating the dynamic speed compensation process above, Figure 13 shows the arbitration factor for the robot autonomy from the speed shared controller in a normalized time scale. The red curve represents the averaged value across trials, and the range of standard deviations is indicated with a shaded background. As shown in the Figure 13, the robot autonomy provided little assistance in the beginning (the end-effector was far away from the target object) as the certainty of system-inferred user intention for reaching the target was low. As the reaching task went on, the distance between the end-effector and the target object continuously decreased. Then, the shared controller got more and more confident with the user’s goal, and the arbitration factor for the robot autonomy thus gradually outweighed that for the user, and the robot autonomy dominated the control with a full speed for moving toward the target.

## Discussion

### The Proposed Hybrid Gaze-BMI

To potentially assist individuals suffering from severe motor impairments of upper limbs, the development of effective user control of dexterous robotic assistive manipulators requires intuitive and easy-to-learn-and-use interfaces that produce continuous-valued inputs. In the past decades, invasive BMI approaches have achieved relatively accurate and continuous control of a robot up to 10 DoFs 0. However, the surgical risks associated with current invasive BMIs may outweigh the advantages of effective robotic arm control. The proposed non-invasive and hybrid Gaze-BMI may provide an alternative solution that diminishes medical risks, at the costs of reduced control accuracy and number of controlled DoFs.

In general, the proposed hybrid gaze-BMI operating in the continuous-velocity mode is intuitive and easy-to-learn-and-use. The gaze-tracking system can be calibrated and proficiently driven by a user with no previous experience within 1 min, while maintaining sufficient precision in specifying the movement direction for the end-effector intuitively. Since the input from pure gaze-tracking might be free of intent, it is complemented with an intentional continuous-valued speed input from the 2-class BMI, whose calibration usually did not exceed 5 min. After a short familiarization, all the users could constantly input the movement direction for the end-effector with the gaze-tracking modality, while simultaneously regulating the speed of the end-effector with the BMI modality.

Many studies have utilized the BMI to direct the assistive robot and wheelchairs for a potential population of patients who suffer from severe impairment in upper limbs. Compared with their adopted synchronous BMI or asynchronous BMI, which can only produce discrete-valued velocity commands for the assistive devices, the combination of gaze-tracking and BMI in our work provides users with a flexible HRI for volitionally moving the end-effector continuously and freely on a horizontal plane. Such a continuous-valued velocity (movement direction and speed) control advantage means that not only the end-effector could follow natural movement paths determined by the user in real-time (rather than following predefined ones, Rebsamen et al., 2010; Zhang et al., 2017), but also that the end-effector is always moving as long as it is volitionally directed by the user during the horizontal reaching task. By contrast, this is not the case, in particular, for the synchronous (P300, SSVEP-based) BMI-controlled robotic arms or wheelchairs, where the assistive devices have to spend a lot of time idle, waiting for inputs from the user.

### The Proposed Shared Control Paradigms

According to the online experimental results in section “Results,” on average, 34% trials failed to prevent the end-effector from colliding with the obstacles with the **MC** paradigm (see Table 2). Moreover, such a paradigm also yielded the longest average EETL and CT across trials and subjects (refer to Figures 8, 11). This is largely due to the significant inherent difficulties for the pure hybrid Gaze-BMI control. For instance, one of the difficulties is that it requires a swap of the mental statuses (e.g., rest or hand motor imagery) in time so as to delicately regulate the speed of the end-effector when approaching the target or avoiding the obstacles. However, evoking a desired mental command required effort from the user and, sometimes, multiple attempts. Such difficulty was further exaggerated by the use of the consumer-level EPOC hardware and the unsatisfying performance of the software for online MI state detection. Thanks to the application of the shared control in both the movement direction and speed, the **SC***_*DS*_* paradigm reduced the task difficulty by adding autonomous supportive behavior to the system. Ultimately, it resulted in 100% SRR, satisfying average EETL and the shortest average CT across trials and subjects (refer to Figures 8, 11).

Furthermore, for the proposed shared control paradigms during the horizontal reaching task, the control for the end-effector always reflected the simultaneous input from both the user and the robot autonomy by a dynamic linear blending (arbitration). In this way, the paradigms allow the user to directly control the majority of the movement, while smoothly increasing the assistance from robot autonomy during the most difficult parts (e.g., collision avoidance, target approaching) of the task for the user, reaching a balance between the user’s perceived control and the reliable assistance provided by the robot autonomy. By contrast, for the existing studies that apply the shared control strategies in robotic arm, wheelchair, and mobile robot systems driven by the non-invasive HRI, the user commands and the robot autonomy commands switch either in the beginning of the reaching task (i.e., the robot autonomy takes over to finish the remaining routine, triggered by the user) (McMullen et al., 2014; Chen et al., 2019) or during the most difficult parts of the reaching task (i.e., the robot autonomy takes over to finish sub-tasks requiring a high precision, triggered by the user) (Zhang et al., 2017; Xu et al., 2019). Such a switching characteristic makes the user clear that the system is providing the autonomous control, which is known to yield reduced sense of agency and frustration for the user according to previous user studies (Kim et al., 2006; Dragan and Srinivasa, 2013; Muelling et al., 2017).

### Limitations and Future Work

This work has presented a proof-of-concept implementation for new shared control paradigms that could potentially help to better integrate the robot autonomy in assistive robotic arm manipulation applications while keeping the user in control with a novel HRI as much as possible. In particular, the current study focus was set primarily on the horizontal reaching task since strategies for maintaining the user in control to the largest extent during other operations (e.g., grasping, lifting, etc.) were presented in our previous paper (Zeng et al., 2017). These studies together may open up possibilities for sophisticated scenarios.

The currently implemented hybrid gaze-BMI is just one of the many systems components that will be improved in future developments. One of the future works will be devoted to re-implementing the detection of the mental state related to motor imagery using a medical EEG acquisition system. Another future study will extend the current 2D gaze tracking into 3D one with a wearable eye-tracker as in Abbott and Faisal (2012) and Li S. et al. (2017) In this way, the 3D coordinates of the gaze in the 3D environment can be estimated for generating the movement direction commands, by which the true 3D continuous motion of the end-effector can be achieved during reaching and without decomposing it into a horizontal 2D motion and a vertical motion sequentially. In addition, the infrequent switching between tasks (e.g., from reaching to grasping, from grasping to delivering, from delivering to releasing, etc.) was implemented automatically. Future studies will investigate advanced BMI classification methods for recognizing multiple mental states, in order to trigger the task switching. For this aim, enhanced visual or haptic cues about the state of task execution by the robot can be further provided to the user in order to increase usability and transparency of the system as in our previous work (Zeng et al., 2017).

To extend the proposed proof-of-concept semi-autonomous robotic system for performing tasks in realistic environments, the currently used stereo-camera will be replaced with depth sensors. Besides, an advanced computer vision module will be employed to provide more effective object perception and modeling for the robot.

The shared control paradigms in the current study were designed based on the environmental context only, and the same paradigms were applied for each participate throughout the task. In future, the personalized shared control paradigms will be developed, where the paradigms adapt to the user’s evolving capability and needs given not only the environmental context but also the state of the user. This may allow the user to use intelligent assistive devices in their day-to-day lives and for extended periods of time.

## Conclusion

This paper presents a semi-autonomous robotic system for performing the reach-and-grasp task. In particular, we propose a new control paradigm for the robotic arm reaching task, where the robot autonomy is dynamically blended with the gaze-BMI control from a user. Meanwhile, the hybrid gaze-BMI constitutes an intuitive and effective input through which the user can continuously control the robotic arm end-effector moving freely in a 2D workspace with an adjustable speed proportional to the user’s motion intention strength. Furthermore, the presented shared control paradigm allows the user to directly control the majority of the movement while smoothly increasing the assistance from robot autonomy the during the most difficult parts (e.g., collision avoidance, target approaching, etc.) of the task for the user, reaching a balance between the user’s perceived control and the reliable assistance provided by the robot autonomy. The experimental results demonstrate that the proposed semi-autonomous robotic system yielded a continuous, smooth and collision-free motion trajectory for the end-effector approaching the target. Compared to the system without the assistance from robot autonomy, it significantly reduces the rate of failure as well as the time and effort spent by the user to complete the tasks.

## Data Availability Statement

The datasets generated for this study are available on request to the corresponding author.

## Ethics Statement

The studies involving human participants were reviewed and approved by the Ethics Committee of Southeast University. The patients/participants provided their written informed consent to participate in this study.

## Author Contributions

HZ and YW designed the study. YS and XH set up the experiment platform. BX and YW performed the experiment. HZ, YS, and YW analyzed the data and wrote the manuscript. AS, HL, and PW were involved in critical revision of the manuscript. All authors read and approved the final manuscript.

## Conflict of Interest

The authors declare that the research was conducted in the absence of any commercial or financial relationships that could be construed as a potential conflict of interest.
